# Genetic and Epigenetic Mechanisms Underlying Reversible Adaptive Responses in Fungi

**DOI:** 10.3390/jof12050309

**Published:** 2026-04-23

**Authors:** Lufeng Dan, Siyin Liu, Zhihao Qiang, Xiaowen Ye, Jinping Zhang

**Affiliations:** Antibiotics Innovation and Resistance Control Key Laboratory of Sichuan Province, School of Pharmacy, Chengdu University, Chengdu 610106, China; 15883620097@163.com (S.L.); 15228422935@163.com (Z.Q.); 18388124690@163.com (X.Y.); yolo_61@163.com (J.Z.)

**Keywords:** fungi, adaptive responses, genetic mechanisms, epigenetic regulation, phenotypic plasticity

## Abstract

The remarkable ecological success of fungi is supported by their capacity for rapid and often reversible molecular responses to fluctuating environments. While conventional evolutionary theory has largely emphasized mutation and selection as central drivers of adaptation, many environmentally responsive fungal traits are also shaped by molecular processes that generate reversible phenotypic variation on ecological or developmental timescales. This review synthesizes current knowledge on reversible genetic and epigenetic mechanisms underlying fungal phenotypic plasticity by integrating insights from programmed genetic rearrangements such as mating-type switching, transposable element activity, variation in tandem repeats and the behavior of accessory chromosomes, together with dynamic epigenetic processes including histone modifications, DNA methylation, chromatin remodeling and RNA mediated regulation. Together, these mechanisms form an interconnected framework that enables rapid and, in many cases, reversible phenotypic diversification, although their consequences range from transient regulatory shifts to partially or fully irreversible sequence-level changes. We highlight the molecular machinery that governs reversibility and its evolutionary implications for fungal pathogenesis, symbiosis, and biotechnology. By uniting genetic and epigenetic perspectives, this review advances a holistic framework in which reversibility is treated as a key property of fungal phenotypic plasticity, helping fungi balance stability with flexibility under environmental challenge. Understanding these mechanisms provides new insights into fungal evolution, and opens new avenues for antifungal intervention and the rational design of industrially valuable fungal strains.

## 1. Introduction

The fungal kingdom exemplifies evolutionary success, with its members colonizing virtually every ecological niche on earth, from extreme environments to human hosts [1,2]. This success is closely associated with their capacity to respond to environmental fluctuations, such as shifts in temperature, nutrient availability, pH, and exposure to antimicrobial compounds [3]. For decades, the predominant framework for understanding adaptation has emphasized genetic mutations as the central mechanism, referring to random alterations in DNA sequences that become fixed through natural selection. Importantly, although many mutations, including point mutations, are reversible at the molecular level, their occurrence and fixation are largely stochastic rather than directed by environmental demand. While undeniably the engine of long-term evolution, this model has inherent limitations in explaining rapid and often transient responses to environmental stresses. The stochastic occurrence of mutations, together with their often deleterious or context-dependent consequences and the absence of a regulated route back to a previous genotype under changing environmental conditions, makes mutation-based adaptation comparatively slow on short timescales [4]. In dynamic environments, a mutation that confers a selective advantage at one point in time may later become detrimental [5]. This raises a broader biological question: how can fungi generate rapid, flexible, and reversible phenotypic responses to fluctuating conditions without relying exclusively on permanent genetic alterations?

To cope with the limitations of permanent sequence change on short timescales, fungi employ a broad range of molecular processes that underlie reversible phenotypic plasticity. These processes do not constitute adaptation per se; rather, they generate transient phenotypic variation that may subsequently be shaped by natural selection. Through these mechanisms, a fungal cell can access different phenotypic states in response to environmental cues and, in many cases, return to a previous state when conditions normalize. Reversible processes provide a dynamic layer of regulation that can fine-tune gene expression and cellular function over timescales ranging from minutes to multiple generations of cell division. This phenotypic flexibility is relevant to fungal fitness and underpins processes such as host invasion, immune evasion, biofilm formation, metabolic versatility, and developmental transitions [5,6,7,8]. In this context, reversibility should be understood as a property of molecular processes that enables dynamic transitions between phenotypic states under fluctuating environmental conditions. This perspective highlights that reversibility can influence fitness under fluctuating conditions without implying that the underlying molecular process is equivalent to adaptation itself.

These reversible mechanisms can be broadly divided into two interrelated categories, consisting of reversible genetic modifications and reversible epigenetic modifications [9,10]. Reversible genetic mechanisms include dynamic genome structural variations and mobile genetic elements that modulate gene function and generate phenotypic variability. These mechanisms, including transposable elements, tandem repeats, genome rearrangements, and accessory chromosomes, are discussed in Section 2. Reversible epigenetic regulation involves dynamic modifications of chromatin and gene expression, including DNA methylation, histone modifications, chromatin remodeling, and RNA-mediated processes. These mechanisms provide rapid and often reversible control of transcriptional states and are described in Section 3. Importantly, reversibility should be viewed as a continuum rather than a binary property, as some molecular states can be readily reversed whereas others may have persistent or irreversible sequence-level consequences.

Genetic and epigenetic mechanisms have traditionally been viewed as operating largely independently. This review synthesizes recent advances in the molecular machinery underlying these reversible systems and examines the crosstalk between genetic and epigenetic pathways, including how epigenetic states may influence the accessibility of genomic regions to genetic rearrangement. Compared with previous reviews that have focused primarily on either genetic or epigenetic mechanisms in isolation, the novelty of this review lies in integrating both perspectives within a unified framework centered on reversibility and phenotypic plasticity. Furthermore, beyond their evolutionary implications, these processes are increasingly being explored in biotechnology, including fungal strain improvement, modulation of secondary metabolism, activation of silent biosynthetic pathways, and the development of antifungal strategies. By integrating these concepts, this review aims to clarify how dynamic molecular processes contribute to the ecological success and functional versatility of fungi, while also highlighting their relevance to antifungal intervention and fungal biotechnology.

## 2. Reversible Genetic Changes

Reversible genetic changes in fungi are not uniformly distributed across the kingdom and should be interpreted in a species- and lineage-specific manner [11,12]. The mechanisms discussed below have been characterized predominantly in ascomycetes, whereas comparable or functionally analogous processes have been reported more sporadically in basidiomycetes. To preserve biological context, each mechanism is discussed together with representative fungal species, its phylogenetic distribution, and, where possible, its relevance to parasitic, commensal, or mutualistic interactions; fungal development; and biotechnological exploitation. In addition to mini-chromosomes, transposable elements, mating-type switching, and tandem repeats, other mechanisms contributing to reversible or transient genome-associated phenotypic variability include repeat-induced point mutation (RIP), methylation induced premeiotically (MIP), heterokaryon formation, prion-based inheritance, and RNA-level mutational or editing-like processes (Table 1). Although these mechanisms differ in their molecular logic and degree of reversibility, together they illustrate that fungal phenotypic flexibility often relies on dynamic genome-associated systems rather than exclusively on fixed mutation.

### 2.1. Mini-Chromosomes and Supernumerary Chromosomes

Mini-chromosomes and supernumerary chromosomes are classified as conditionally dispensable chromosomes, as they are not required for essential vegetative growth, such as under laboratory conditions. Mini-chromosomes are typically less than 2 Mb in size and are frequently derived from core chromosomes through horizontal transfer, segmental duplication, or the stabilization of dispensable genomic regions [13]. Supernumerary chromosomes, or B chromosomes, are dispensable chromosomal elements that exist outside the standard A complement; unlike mini-chromosomes, which are defined by reduced size, B chromosomes are characterized by their independent chromosomal identity and non-essential status rather than by length [14,15]. Although initially considered genetically inert, accumulating genomic and transcriptomic evidence indicates that these chromosomes may harbor intact protein-coding genes, gene fragments, regulatory elements, and transposable elements [16]. Notably, comparative analyses across species have shown that B chromosomes often contain intact genes involved in cell cycle regulation, recombination, and metabolism, while exhibiting either transmission advantages or selective loss [17]. In addition, accessory chromosomes exhibit distinct epigenetic and chromatin features, including enrichment in repetitive sequences, reduced gene density, heterochromatic modifications, and altered centromere and kinetochore organization [18,19]. Supernumerary chromosomes frequently display atypical inheritance patterns, such as meiotic and mitotic drive, somatic mosaicism, variable copy number variation, and, in some lineages, elimination across generations [20,21]. These characteristics reflect their pronounced meiotic and mitotic instability relative to core chromosomes.

**Figure 1:**
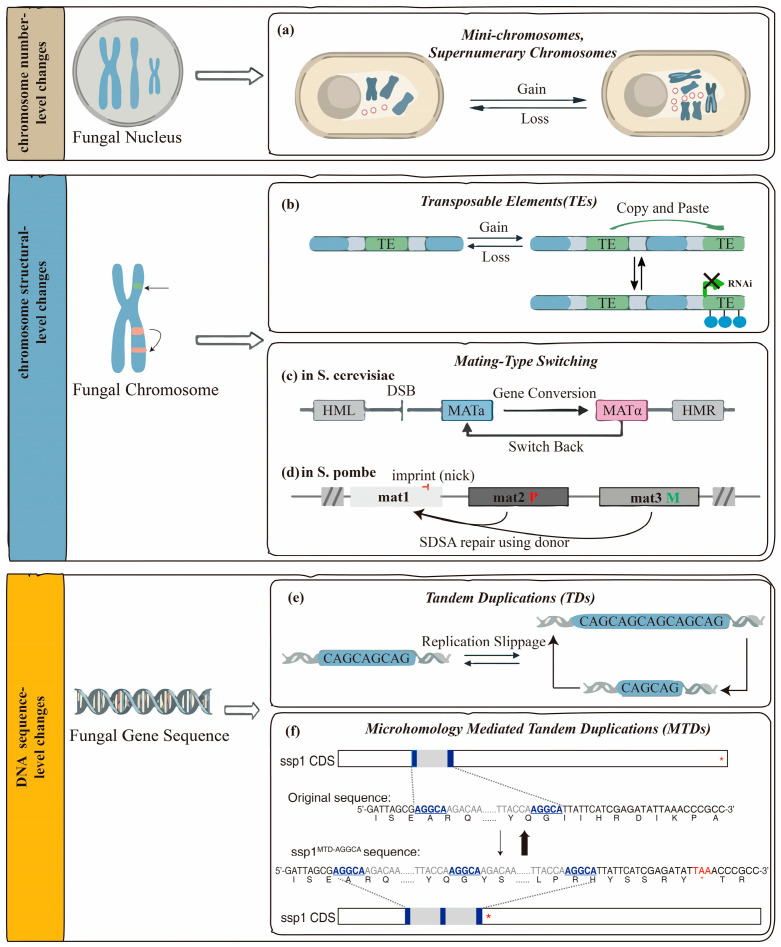
Schematic overview of major genetic mechanisms generating reversible variation in fungi, spanning chromosome number, chromosome structure, and DNA sequence levels. (**a**) Gain and loss of mini-chromosomes or supernumerary chromosomes in fungal nuclei. Red circles represent supernumerary chromosomes, which are non-essential, accessory genomic elements that can vary in presence and copy number. (**b**) Chromosome structural-level changes mediated by transposable elements (TEs). TEs contribute to genome restructuring through insertion, excision, and copy-and-paste mechanisms, resulting in TE copy number variation. Host genome-defense pathways, such as RNA interference (RNAi) and heterochromatin (the blue circle: H3K9me2 modification), act to restrict TE proliferation and promote genomic stability. (**c**) Mating-type switching in *Saccharomyces cerevisiae*. The mating-type switching is mediated by a programmed double-strand break (DSB) at the MAT locus, followed by gene conversion using one of the silent donor cassettes (HML or HMR). This process enables reversible switching between MATa and MATα cell types. (**d**) Mating-type switching in *Schizosaccharomyces pombe*. The mating-type switching is initiated by a site-specific imprint at the active mat1 locus. Repair through synthesis-dependent strand annealing (SDSA) using the silent mat2-P or mat3-M donor loci results in mating-type interconversion. (**e**) Tandem duplications (TDs) at the DNA sequence level. At the DNA sequence level, tandem duplications arise primarily through replication slippage, leading to expansion or contraction of short repeated sequences or gene segments and contributing to copy number variation. (**f**) Microhomology-mediated tandem duplications (MTDs) in the *ssp1* gene [107]. The reversible MTDs occur during DNA repair processes that utilize short microhomologous sequences, resulting in localized duplications within *ssp1* coding regions. Underlined and bold bases indicate microhomology pairs. The pre-matured stop codon is marked with red. The red asterisks denote terminators. (**a**–**e**) are original artwork created by the authors.

The defining properties of these chromosomes, including strain-specific occurrence, genomic instability, and enrichment in adaptive genes, make them efficient mediators of dynamic and potentially reversible genome variation. They allow fungi to acquire, retain, and discard extensive trait-associated gene sets under changing selective conditions (Figure 1a). A representative example is provided by the plant pathogen *Fusarium oxysporum*, in which accessory chromosomes known as pathogenicity chromosomes determine host-specific infectivity [22]. Acquisition of these chromosomes enables rapid host range expansion, whereas their loss may occur when encoded effectors become ineffective or when their maintenance costs reduced fitness, thereby restoring a phenotype closer to that of the accessory-chromosome-free background strain [23,24]. This reversibility reflects genome-level plasticity rather than directed adaptation. In engineered or laboratory systems of *Saccharomyces cerevisiae*, mini-chromosomes can support dosage-dependent expression of introduced or amplified genetic elements and, in some contexts, permit reversible phenotypic modulation through segregation or excision [25,26,27]. These observations are primarily derived from engineered or laboratory contexts, and their relevance to natural populations may be context-dependent. Similarly, reversible aneuploidy-associated phenotypic plasticity has been extensively documented in *Candida albicans*, where chromosome copy-number changes play a central role in transient drug tolerance and stress responsiveness [28,29,30]. Exposure to subinhibitory concentrations of fluconazole induces whole-chromosome aneuploidy, particularly disomy of chromosomes 5 and 7, resulting in upregulation of ergosterol biosynthesis genes and increased drug resistance. Upon drug withdrawal, aneuploid cells frequently revert to euploidy through chromosome loss, restoring cellular fitness [30,31]. Likewise, treatment with brefeldin A induces trisomy of chromosome 3, leading to transient increases in filamentation and virulence that dissipate after stress removal [28,32,33].

The stability and transmission of mini-chromosomes are influenced by both genetic and epigenetic determinants. In particular, chromatin state plays a critical role in regulating chromosome stability. A repressive heterochromatic environment may suppress deleterious genetic elements while simultaneously increasing chromosomal instability. Accordingly, alterations in the epigenetic landscape can modulate the probability of chromosome silencing or loss [34,35]. In addition, the centromeres of accessory chromosomes frequently diverge from those of core chromosomes and are often structurally weaker or epigenetically unstable, which predisposes them to mis-segregation during cell division and facilitates chromosome loss. Furthermore, the high density of transposable elements on these chromosomes promotes diversification through ectopic recombination, generating novel chromosomal variants that can be subjected to natural selection [34,36].

Beyond pathogenic interactions, accessory chromosomes may also contribute to ecological flexibility in non-pathogenic settings by modulating secondary metabolism and niche-associated regulatory programs. Such genome-level plasticity is likely relevant to endophytic persistence and mutualistic interactions, where reversible adjustment of host- and niche-associated traits may be advantageous [37,38,39]. From an applied perspective, mini-chromosomes and accessory chromosomes are increasingly considered useful platforms for chromosome engineering, modular gene stacking, and strain improvement in fungal biotechnology. Their instability may also intersect with developmental transitions, including sporulation or stress-associated morphogenetic switching, by altering dosage-sensitive regulators of differentiation.

Mini-chromosomes and supernumerary chromosomes may represent a genome-level source of phenotypic variability associated with fungal plasticity. They appear to function as modular and exchangeable genetic elements that can be rapidly acquired or eliminated, thereby potentially conferring a degree of phenotypic plasticity that would be difficult to achieve through the gradual accumulation of mutations within the core genome. Consequently, these chromosomes enable rapid phenotypic shifts under changing environmental conditions, although such changes are not necessarily adaptive in all contexts. Future studies, particularly those integrating long-read sequencing technologies and live-cell imaging approaches, are expected to elucidate the mechanisms underlying their segregation, stability, and gene regulation. Such efforts will provide deeper insights into fungal genome dynamics and may identify novel targets for the management of pathogenic fungi. In species in which accessory chromosomes carry host-interaction genes, such as *Fusarium oxysporum*, their gain or loss can directly alter parasitic fitness by modulating host specificity and virulence [22,23]. In endophytic or mutualistic settings, analogous chromosome-linked shifts may reprogram niche-associated traits, including colonization-related, metabolic, or stress-responsive gene expression. Accessory-chromosome instability may also intersect with fungal development, as copy-number changes during cell-type transitions could alter the dosage of morphogenetic regulators under environmental stress. From a biotechnological perspective, the modularity of dispensable chromosomes makes them attractive platforms for chromosome engineering, pathway stacking, and reversible optimization of strain performance without extensive modification of the core genome.

### 2.2. Transposable Elements

Transposable elements (TEs) are now recognized as major drivers of fungal genome plasticity, influencing gene regulation, chromosome architecture and genome evolution [40,41]. The dynamic and stress-responsive regulation of many fungal transposable element families by epigenetic and small RNA-mediated mechanisms highlights their role as an important source of phenotypic variability and regulated genome plasticity [42,43]. Recent work in fungal plant pathogens, mutualists and human pathogens shows that (i) population-level TE invasions can remodel genome size and gene content over decades [44]; (ii) stress-induced TE derepression alters virulence and fungicide resistance [45]; and (iii) epigenetic silencing and genome-defense pathways, like DNA methylation, RNA interference, and repeat-induced point mutation, continually reset TE activity toward a new equilibrium [46].

Transposable elements are characterized by terminal inverted repeats or long terminal repeats flanking protein-coding regions that encode enzymes such as transposase or reverse transcriptase and integrase, which catalyze transposition and often result in the formation of target site duplications in the host genome [47]. Through large-scale comparative studies, transposable elements have been recognized as important components shaping genome architecture in fungi [40]. Comparative surveys across 625 fungal genomes indicated that ancient and fragmented transposable elements are often integrated into coding regions, while younger, potentially active copies are enriched in repeat-rich genomic compartments that are largely devoid of essential genes. Analyses of 18 fungal genomes have shown that variation in transposable element content is closely linked to genome-wide structural organization and transcriptional profiles, with enrichment in accessory chromosomes and variable chromosomal arms [48]. Studies of plant pathogenic fungi indicate that effector genes reside in transposable element-rich genomic compartments characterized by rapid evolution, frequent structural variation, and extensive gene gain and loss, with enrichment for genes involved in host interaction and host-associated functions [40,49]. Overall, transposable element activity promotes genomic plasticity that enables recurrent reorganization and refinement of allelic and regulatory variation, especially those encoding effectors and resistance factors, and serves as a dynamic source of genetic variation that may be subject to natural selection.

These patterns have been described across multiple fungal taxa, including ascomycetes such as *Magnaporthe oryzae* and *Zymoseptoria tritici*, as well as basidiomycetes, although the distribution and activity of transposable elements vary substantially among lineages. Transposable element mobilization affects gene function and regulation via several mechanisms, such as insertional mutagenesis, genome rearrangement, and regulatory network modification, contributing to the generation of variation subject to selective processes. Insertions of transposable elements into coding or regulatory regions may disrupt or modify gene expression and can give rise to gain-of-function or loss-of-function mutations [43,50]. Recombination events between non-allelic copies of the same transposable element distributed across the genome can generate chromosomal inversions, deletions, and duplications, and in some cases lead to the emergence of accessory chromosomes. Transposable element insertions within promoter regions can modify local regulatory landscapes by contributing transcription factor binding sites, which can lead to the emergence of new gene expression responses to environmental or developmental cues [48,50,51]. In *Magnaporthe oryzae*, transposable element-mediated processes contribute to diversification of avirulence effector genes, supporting rapid evolutionary dynamics and the circumvention of host resistance [52,53]. Transposable element insertions and excisions may generate reversible changes in gene expression and regulatory networks, allowing organisms to rapidly respond to environmental or developmental changes, with the potential for reversion under specific genomic or regulatory conditions (Figure 1b).

To maintain genomic integrity, fungi have evolved robust mechanisms to repress transposable element activity. Under conditions in which a transposable element insertion becomes deleterious or selective pressure subsides, the epigenetic machinery responsible for transposable element repression can also be restored. Through the RNA interference pathway, small interfering RNAs homologous to active transposable elements are generated and promote the deposition of repressive histone and DNA methylation marks, resulting in heterochromatin formation and suppression of transposable element activity [46,54,55]. RNA interference-mediated heterochromatin formation enables efficient transcriptional silencing of transposable elements (Figure 1b). As a consequence, even when a transposable element insertion persists at the DNA level, its impact on gene expression can be neutralized, effectively concealing genetic variation and permitting reversion to a prior phenotypic state [46]. Moreover, precise excision of specific DNA transposons can also lead to genuine genetic reversion [56]. The interplay between stress-induced transposable element activation and subsequent re-silencing under homeostatic conditions gives rise to a dynamic equilibrium that modulates genetic diversity and phenotypic outcomes.

In fungi, transposable elements constitute a dynamic and tightly regulated source of genome variation. Their biological consequences are strongly species- and context-dependent: in parasitic fungi, accessory chromosomes frequently restructure effector-rich compartments and host- or stress-responsive loci, whereas in endophytic or mutualistic fungi they may contribute more to metabolic plasticity and niche responsiveness [37,57,58]. Because TE activity can be re-silenced after stress relief, these systems are particularly relevant to transient phenotypic diversification, developmental transitions such as conidiation and sporulation, and biotechnological applications including insertional mutagenesis, genome diversification, and activation of silent biosynthetic pathways.

### 2.3. Mating-Type Switching

A range of sexual reproductive strategies have evolved in fungi to accommodate environmental variability, with mating-type switching functioning as a regulated and reversible genetic process [59,60]. Mating-type switching, which is predominantly observed in ascomycete yeasts, enables a single haploid cell to produce progeny of opposite mating types through programmed genomic rearrangements. This process facilitates self-fertilization without the necessity of a compatible mating partner [61]. It involves the alternate replacement of a small segment of specific genomic DNA with another, without the permanence associated with random mutations. This results in the substitution of the master regulatory genes in the mating pathway, leading to a switch in cell type and mating behavior [62]. This self-mating capability promotes transient homothallism in lineages typically predisposed to heterothallism. It constitutes a reversible genetic system that transiently alters reproductive compatibility without permanently changing genome content; any adaptive consequence depends on ecological context and subsequent selection rather than on the switching event itself [63].

This mechanism has been primarily characterized in ascomycete yeasts such as *Saccharomyces cerevisiae* and *Schizosaccharomyces pombe*, whereas comparable systems are less well defined in basidiomycetes, highlighting lineage-specific differences in mating regulation. The molecular mechanism underlying mating-type switching has been most comprehensively characterized in *Saccharomyces cerevisiae*, where it follows a well-established cassette model. This system comprises an active MAT locus flanked by two transcriptionally silent mating-type cassettes, HML (MATα) and HMR (MATa), which serve as genetic reservoirs [62,64]. Mating-type switching is initiated by a site-specific double-strand break (DSB) at the MAT locus, generated by the HO endonuclease that is transiently expressed in mother cells during the G1 phase of the cell cycle [62,65]. Transcriptional silencing mediated by the Sir2/3/4 protein complex protects the silent HML and HMR loci from HO-induced cleavage, thereby preserving their integrity [62,66]. The DSB at the MAT locus is repaired through homologous recombination (HR), using one of the silent cassettes (HMLα or HMRa) as the donor template [67]. Donor choice is non-random, with MATa cells preferentially utilizing HMLα as the repair template, and vice versa. This repair process proceeds via non-reciprocal gene conversion, resulting in replacement of the MAT sequence and a switch in mating type from a to α (Figure 1c) [68,69]. Importantly, the silent donor loci remain unaltered, allowing mating-type switching to occur reversibly across successive generations. Expression of HO is stringently regulated and restricted to mother cells, ensuring that each switching event produces a pair of daughter cells with opposite mating types, thereby facilitating mating [64,68]. Under conditions of nitrogen limitation, repression of HO expression is alleviated, leading to mating-type switching in approximately 60% of the cell population. This increased switching frequency promotes self-diploidization followed by sporulation, a reproductive response associated with increased spore viability under nutrient-limited conditions [70,71,72]. In industrial contexts, particularly in brewery strains, structural rearrangements such as inversions at the MAT locus have been shown to increase ethanol tolerance by promoting the formation of switched diploids. Subsequent loss-of-heterozygosity (LOH) events enable the re-emergence of haploid cells after fermentation, thereby optimizing clonal foraging while minimizing inbreeding depression [73,74].

In *Schizosaccharomyces pombe*, the mating-type region includes the active mat1 locus flanked by the silent mat2-P and mat3-M cassettes, with donor choice regulated by mat2-Pt and mat3-Mt [75,76]. Mating-type switching is replication-coupled, involving a programmed imprint or single-strand nick at the mat1 locus, rather than a double-strand break (DSB) [77,78]. This imprint halts replication, and during the next cell cycle, the replication fork encountering the imprint collapses, forming a DSB. The DSB is repaired via synthesis-dependent strand annealing (SDSA) using mat2-P or mat3-M as templates (Figure 1d). The donor cassette is selected deterministically based on the mat1 allele and replication fork direction, resulting in a stereotyped switching pattern (e.g., P cells switch to M, and M cells switch to P). The imprint, if not resolved into a DSB, can be faithfully repaired [75]. Silent loci are protected by a heterochromatin barrier, mediated by the heterochromatin binding protein Swi6, which enforces unidirectional recombination. Clr4-mediated erasure propagates this tolerance through mitosis before meiotic resetting [76,79]. Similar regulatory principles have been proposed in other fungal species, including *Candida* auris, where mating-type-like regulatory mechanisms have been associated with phenotypic variation and antifungal responses [80].

Mating-type switching, underpinned by both programmed DNA rearrangement and epigenetic regulation, is best understood as a lineage-enriched mechanism of reproductive flexibility rather than a universally conserved fungal trait. In ecological terms, this mechanism can provide an advantage in parasitic or host-associated niches where compatible partners are limiting, because switching enables reproductive assurance and subsequent sporulation. It may also support persistence in fluctuating environments by coupling self-diploidization, meiotic entry, and stress-resistant spore formation. These developmental consequences are especially relevant to nutrient limitation and other stress conditions that favor sporulation as an adaptive dispersal strategy. From an applied perspective, mating-type switching systems have been exploited in yeast genetics and strain construction, and they remain valuable tools for controlled crossing, genome manipulation, and industrial breeding [68,81,82].

### 2.4. Tandem Repeats

Tandem repeats (TRs) comprise head-to-tail arrays of identical or near-identical sequence motifs and include microsatellites with repeat units of 1 to 6 base pairs and minisatellites with units of 10 to 60 base pairs, spanning from a few to thousands of repeats [83]. The intrinsic instability associated with dynamic copy number variation is not solely a source of genetic noise but may represent a finely regulated mechanism contributing to reversible phenotypic variation [84,85]. In fungal genomes, tandem repeats account for approximately 1% to 5% of the total DNA content and are predominantly enriched in subtelomeric regions, introns, and coding sequences, where they function as mutational hotspots owing to their high susceptibility to replication slippage and recombination [86,87]. By acting as adjustable genetic elements, these repeats enable fungi to rapidly regulate gene expression and protein function in response to changing environmental conditions, thereby providing a rapid source of genetic and phenotypic variability distinct from conventional mutational mechanisms.

The high mutation rate of TRs, which is often orders of magnitude greater than point mutations, stems from DNA polymerase slippage during replication, where the nascent strand misaligns with the template, leading to repeat-unit gains and losses (Figure 1e) [88,89]. DNA repair pathways, particularly those mediated by microhomology, further contribute to alterations in copy number and the generation of complex genomic rearrangements [90,91]. Under conditions of replication stress, microhomology-mediated end joining is preferentially engaged at tandem repeat loci, where it exploits short homologous sequences within the repeats to mediate error-prone repair, frequently resulting in repeat expansions or contractions. In contrast, high-fidelity homologous recombination contributes to the maintenance of tandem repeat stability. The relative engagement of these repair pathways is modulated by stress-responsive factors, including the Mre11-Rad50 complex, thereby establishing a regulatory framework that governs tandem repeat-mediated variation [92,93]. In addition, some tandem repeat sequences can form secondary DNA structures that impede replication fork progression, thereby promoting translesion synthesis, replication fork reversal, and microhomology-mediated template switching, all of which contribute to repeat instability. Replication-associated factors such as Mrc1 and Tof1, together with transcription-induced RNA DNA hybrid formation, further modulate replication dynamics and genome stability at tandem repeat loci [94,95].

Accumulating evidence indicates that tandem repeat instability is tightly regulated by epigenetic mechanisms and stress-responsive signaling pathways [96]. Repressive chromatin modifications, including histone H3 lysine 9 trimethylation and DNA methylation, have been shown to promote tandem repeat stability by restricting DNA accessibility and suppressing recombination frequency [97,98]. In contrast, chromatin remodeling induced by environmental stress, such as increased histone acetylation, can transiently relax chromatin structure, thereby enhancing tandem repeat instability and promoting copy number variation. This form of epigenetic plasticity is thought to enable fungi to link environmental sensing with regulated genetic diversification [96,99,100]. The selection of DNA repair pathways plays a central role in determining tandem repeat stability. In addition, non-coding RNAs derived from tandem repeat loci have been reported to direct epigenetic silencing complexes, including RNA interference machinery in *Neurospora*, to homologous genomic regions, thereby establishing sequence-specific feedback mechanisms that contribute to the maintenance of defined tandem repeat lengths [101,102]. The mitogen-activated protein kinase signaling pathway has been shown to phosphorylate components of the replication and DNA repair machinery in response to osmotic or oxidative stress, thereby modulating their activity at tandem repeat loci [103,104]. Nutrient limitation mediated by target of rapamycin signaling has been reported to influence the expression of chromatin-modifying enzymes, whereas DNA damage response pathways promote the activation of components involved in microhomology-mediated end joining [105,106]. This mechanism allows fungi to fine-tune tandem repeat instability rates in response to environmental stresses.

Microhomology-mediated tandem duplication (MTD) functions as a precise mechanism for reversible gene dosage variation by generating two tandem copies from a single genomic segment through replication-based repair processes [90,107,108]. The process is initiated when DNA replication stalls or collapses at a specific genomic region, resulting in the exposure of microhomologous sequences. During the repair process, the broken DNA end anneals to these microhomologous sites on the sister chromatid or homologous chromosome, bypassing accurate DNA synthesis and leading to the duplication of the segment between the microhomology sites [90,92,109]. This results in the formation of a direct tandem repeat flanked by microhomologous regions. Importantly, these repeats are inherently unstable. On one hand, homologous recombination between the repeats can efficiently reverse the duplication, thereby restoring the single-copy state [110]. This cycle of stress-induced amplification and growth-dependent reversion allows fungi to dynamically adjust gene dosage in response to environmental pressures, as demonstrated in adaptive traits like drug resistance (Figure 1f) [107]. On the other hand, once a two-copy tandem duplication has formed, the duplicated microhomology units provide repeated substrates for additional rounds of microhomology-mediated slippage, break-induced replication, or template switching, thereby enabling stepwise conversion of a two-copy structure into higher-order multi-copy arrays [90,111]. These expanded repeats enable rapid and reversible modulation of gene dosage, contributing to phenotypic flexibility under dynamic environmental conditions.

These mechanisms have been described mainly in ascomycetes, particularly in model yeasts and filamentous fungi, although tandem repeat instability and copy number variation are broadly relevant across fungal genomes. In parasitic contexts, repeat-mediated variation can reshape effector-rich compartments and other virulence-associated loci, with consequences for host adaptation, immune evasion, and antifungal responses. In mutualistic, and possibly some commensal, fungi, analogous variability may instead support flexible regulation of host-interaction, stress-responsive, and metabolic functions [49,112,113].

From an applied perspective, tandem repeat variation and related genome plasticity mechanisms are highly relevant to fungal biotechnology because reversible modulation of gene dosage and expression can alter metabolite production, secretion capacity, stress tolerance, and strain robustness. Tandem repeat dynamics may also intersect with developmental processes such as conidiation, sporulation, and dimorphic transitions by tuning the expression thresholds of regulatory loci. Thus, tandem repeats function not merely as unstable DNA tracts but as adjustable genetic modules that couple environmental sensing to transient phenotypic diversification.

In summary, tandem repeats function as adjustable genetic modules in fungal genomes, facilitating reversible phenotypic variation through intrinsic instability that is modulated by DNA repair, chromatin state, and, in some systems, RNA-mediated regulation. With increased social challenges due to climate variability and antifungal resistance, these elements provide insights into the molecular mechanisms underlying fungal resilience.

### 2.5. Others

Repeat-induced point mutation (RIP) and methylation induced premeiotically (MIP) are classic fungal genome-defense systems that have been characterized primarily in filamentous ascomycetes. RIP was first characterized in *Neurospora crassa*, where duplicated DNA sequences are recognized during the sexual, premeiotic phase and subjected to extensive C:G-to-T:A transition mutations, thereby inactivating repetitive DNA and restricting transposon proliferation [114,115]. MIP, originally characterized in *Ascobolus immersus*, instead promotes premeiotic cytosine methylation of duplicated sequences and thereby silences repetitive DNA without necessarily generating immediate sequence-level mutation [116,117,118]. Although RIP introduces irreversible point changes at the DNA level, its inclusion in this review is warranted because it helps define the boundary between reversible and irreversible genome-associated responses, particularly in systems where transient repeat activation is followed by lineage-specific genome-defense mechanisms that re-stabilize the genome. By contrast, MIP more directly illustrates reversible or semi-reversible regulation, because methylation states can in principle be remodeled across developmental cycles. These processes are most clearly documented in ascomycetes and have not been shown to operate in the same canonical form across basidiomycetes. Their biological relevance is especially evident in parasitic fungi, where tight control of transposable elements and repeat-rich compartments helps preserve genome integrity while still permitting bursts of innovation in host-interaction loci. More generally, RIP/MIP-like defense systems contribute to long-term genome stability, developmental regulation during sexual reproduction, and the control of sporulation-associated chromatin states. From an applied perspective, RIP has informed fungal forward genetics and mutational analysis, whereas MIP-like methylation phenomena provide conceptual support for reversible epigenetic engineering strategies aimed at silencing repeats or modulating unstable genomic regions.

Heterokaryosis, defined as the coexistence of genetically distinct nuclei within a common cytoplasm, is a common feature of filamentous fungi and is especially characteristic of Dikarya, encompassing both ascomycetes and basidiomycetes [119,120]. This condition provides a rapid route to phenotypic diversification because nuclear ratios can shift under selection, allowing fungal colonies to sample alternative trait combinations without immediately fixing sequence changes in a single genome. In parasitic interactions, heterokaryosis can buffer deleterious alleles, broaden stress tolerance, and facilitate host adaptation, whereas in mutualistic or environmental contexts it may support metabolic complementation and niche persistence. Heterokaryotic states are closely linked to fungal development, including hyphal networking/differentiation, asexual development and sporulation, and parasexual recombination, and can enhance environmental adaptability and stress resilience; in some fungi, they may also facilitate propagation through asexual spores [119,120].

Prion-based inheritance represents a distinct protein-based mechanism of heritable phenotypic change that has been most extensively characterized in the ascomycete yeast *Saccharomyces cerevisiae*. In this system, self-propagating protein conformations can alter translational fidelity and metabolic phenotypes, and in some cases stress-associated traits, across generations without requiring DNA sequence change [121,122]. Because prion states can be both gained and lost, they provide a potentially reversible mode of phenotypic switching that may enhance survival in fluctuating or stressful environments [123,124]. Although canonical fungal prions are best established in ascomycetes, the broader principle of protein-based inheritance expands the conceptual framework of reversible fungal phenotypic switching beyond DNA-centered mechanisms. Biotechnologically, heterokaryosis has long been exploited in strain construction and parasexual genetics, whereas prion biology offers tools for studying protein-based memory and stress-responsive phenotype switching [119,125].

In addition to DNA-based mechanisms, fungi may generate transient phenotypic variability through RNA-level diversification, including RNA editing/transcript recoding processes that alter transcript sequence or quality without establishing stable genomic mutations [126,127]. Compared with canonical DNA mutation, these processes are intrinsically reversible because the underlying genome remains unchanged and altered transcript pools can dissipate rapidly once the inducing condition is removed. Although the mechanistic basis and prevalence of such phenomena remain less well resolved than those of transposons or chromatin-based regulation, transcript-level diversification provides a useful conceptual bridge between reversible genetic and epigenetic change. Such RNA-level plasticity may be particularly relevant during acute environmental stress, host interaction, and developmental transitions requiring rapid response, including conidiation, sporulation, and dimorphic switching. In parasitic settings, transient transcript diversification could expand phenotypic space for immune evasion or drug tolerance; in commensal or mutualistic settings, it may facilitate short-term metabolic reprogramming. From an applied perspective, these mechanisms highlight the possibility of engineering fungal phenotypes through reversible transcriptome modulation rather than permanent genome editing.

### 2.6. Integrative Perspective

Taken together, reversible genetic mechanisms in fungi span a continuum from chromosome-scale variation to repeat dynamics, reproductive locus switching, heterokaryosis, protein-based inheritance, and transcript-level diversification (Table 1). Their phylogenetic distribution is uneven, with many canonical examples established in ascomycetes and fewer mechanistically resolved counterparts in basidiomycetes. Nevertheless, across parasitic, commensal, and mutualistic interactions, these mechanisms converge on a common functional outcome: they expand phenotypic options under fluctuating environments while avoiding immediate commitment to a single permanent genomic state. They also intersect repeatedly with conidiation, sporulation, and dimorphic transitions, emphasizing that reversible genome-associated regulation is tightly integrated with fungal stress biology and life-cycle progression. This same flexibility underlies growing biotechnological interest in chromosome engineering, repeat-based tuning of gene dosage, parasexual strain improvement, and non-permanent manipulation of fungal traits.

## 3. Reversible Epigenetic Changes

Epigenetic mechanisms provide fungi with a particularly important route to reversible phenotypic change because they can remodel transcriptional states without altering the DNA sequence. However, these processes are also strongly species- and lineage-dependent (Table 1). Canonical RNAi-linked heterochromatin formation is best resolved in the ascomycete *Schizosaccharomyces pombe* and in filamentous fungi such as *Neurospora crassa*, whereas DNA methylation systems vary markedly across the fungal kingdom and are absent or highly reduced in some yeasts. For this reason, the mechanisms discussed below are framed together with their representative species, taxonomic distribution, and biological relevance to host interaction, fungal development, and biotechnology.

### 3.1. DNA Methylation and Demethylation

**Figure 2:**
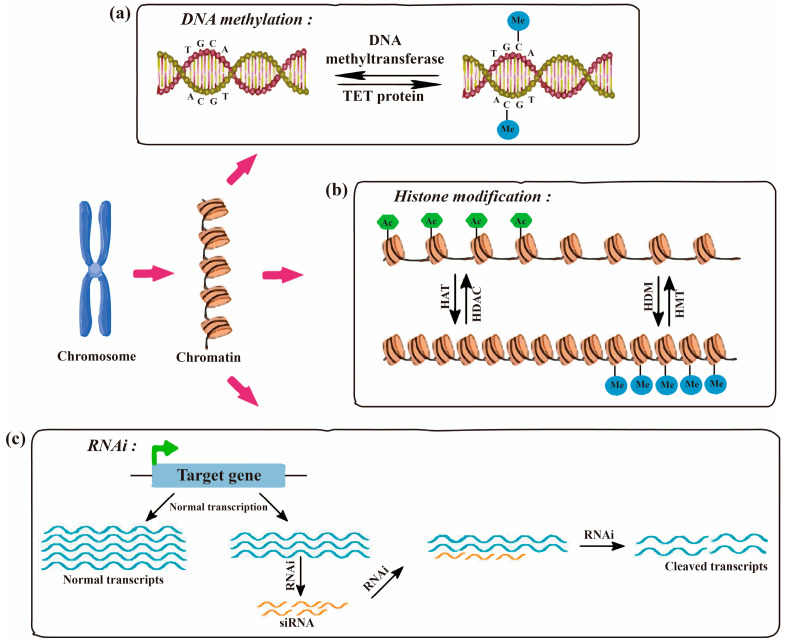
Epigenetic regulatory mechanisms shaping fungal chromatin and gene expression. (**a**) DNA methylation. DNA methylation involves the reversible addition of methyl groups (Me) to cytosine residues, primarily mediated by DNA methyltransferases. Active demethylation can be catalyzed by TET (ten–eleven translocation) proteins. This artwork is original and was created by the authors. (**b**) Histone modification [172]. Post-translational modifications of histones regulate chromatin compaction and transcriptional potential. Histone acetyltransferases (HATs) and histone deacetylases (HDACs) control histone acetylation (Ac), which is generally associated with transcriptionally active chromatin, whereas histone methyltransferases (HMTs) and histone demethylases (HDMs) regulate histone methylation (Me), which is often associated with transcriptionally repressed chromatin. (**c**) RNAi regulates gene expression at the post-transcriptional level [149]. Double-stranded RNA derived from target gene transcripts is processed into small interfering RNAs (siRNAs), which guide sequence-specific degradation of complementary mRNAs, resulting in transcript cleavage and gene silencing.

DNA methylation in fungi represents a conserved epigenetic modification that predominantly occurs at 5-methylcytosine (5mC) and, in certain taxa, at N6-methyladenine (6mA) [128,129]. This modification plays a fundamental role in genome integrity by mediating transposon silencing, regulating developmental processes, and, as increasingly demonstrated, contributing to reversible changes in gene expression and phenotypic plasticity in response to environmental stimuli [128,130]. In many filamentous fungi, such as *Neurospora crassa*, DNA methylation is largely confined to heterochromatic, repeat-rich regions and contributes to silencing of invasive DNA, but the regulation of secondary metabolism requires more investigation [131,132]. By contrast, several yeast species, including *Saccharomyces cerevisiae* and *Schizosaccharomyces pombe*, lack detectable canonical DNA methylation [133]. Notably, certain human pathogenic fungi, such as *Candida albicans* [134,135] and *Cryptococcus neoformans* [136] have retained or independently evolved distinct DNA methylation systems or alternative DNA modifications, which are increasingly implicated in virulence, stress tolerance, and host adaptation. These methylation marks can be dynamically established, maintained, and remodeled in a context-dependent manner, thereby permitting epigenetic states to change without alterations to the underlying DNA sequence. This provides a molecular basis for reversible regulatory responses and phenotypic state transitions (Figure 2a).

DNA methylation does not function as an isolated regulatory mechanism, but instead, interacts extensively with multiple layers of the epigenome, including histone modifications, chromatin architecture, non-coding RNAs, and metabolic signaling pathways [130,131,137,138,139]. For example, histone acetylation generally promotes chromatin relaxation and transcriptional activation by neutralizing the positive charges on histone tails, thereby reducing their affinity for negatively charged DNA. Through these chromatin-based effects, histone modifications can modulate the accessibility of genomic DNA to DNA methyltransferases (DNMTs), consequently shaping DNA methylation landscapes. DNA methyltransferases catalyze the transfer of a methyl group from S-adenosylmethionine to cytosine residues, predominantly within symmetric CpG dinucleotides, but also in non-symmetric sequence contexts [140]. This process is frequently guided by small RNAs or repressive histone modifications, such as histone H3 lysine 9 methylation (H3K9me) [141,142]. Conversely, histone deacetylation is associated with chromatin condensation and transcriptional repression [143,144]. DNA demethylation can occur via either passive or active mechanisms. Passive demethylation results from the failure to faithfully re-establish methylation marks during DNA replication, whereas active demethylation is mediated by enzymatic processes that remove or chemically modify methylated cytosines [145]. Active demethylation is achieved through base excision repair (BER) pathways initiated by demethylases [146]. Although the enzymatic machinery underlying active demethylation has been most extensively characterized in plants and animals, an increasing body of fungal evidence indicates that DNA methylation landscapes are dynamic in response to environmental stress, host-derived cues, and developmental signals, and that partial erasure or remodeling of these patterns underlies reversible modulation of gene expression and phenotype [130,135,147].

Accumulating evidence suggests that some fungi may possess oxidative routes for modifying 5-methylcytosine, and biochemical work has identified a TET homolog in *Coprinopsis cinerea* capable of converting 5mC to 5hmC, 5fC, and 5caC [148]. However, the distribution, physiological relevance, and mechanistic contribution of such pathways to active DNA demethylation across the fungal kingdom remain to be fully established. Subsequent excision of these modified bases and replacement with unmodified cytosine could, in principle, enable reversible loss of DNA methylation, thereby providing a possible mechanistic basis for reactivation of methylation-silenced genes and dynamic transcriptional reprogramming in response to environmental or developmental cues. Consistent with this framework, whole-genome methylome analyses in plant pathogenic fungi, including *Magnaporthe oryzae*, implicate DNA methylation in regulating gene expression programs associated with development and pathogenicity [139]. Infection-stage-dependent modulation of methylation may therefore function as a reversible regulatory switch influencing effector gene expression, with transient demethylation potentially facilitating immune evasion and re-methylation limiting the fitness costs of sustained expression. Similar epigenetic mechanisms have been linked to inducible antifungal tolerance and resistance [149,150], potentially through reversible methylation-associated repression of drug target genes. More broadly, stress-induced remodeling of DNA methylation has been proposed to generate transient phenotypic variability in fungi [7], for example, via transient derepression of transposable elements followed by re-methylation to preserve genome stability. Integration of these epigenetic changes with stress-responsive signaling pathways, such as MAPK and cAMP–PKA cascades [151], may help coordinate reversible epigenetic state transitions during short-term responses to environmental change across limited numbers of generations.

Collectively, these multilayered epigenetic interactions orchestrate dynamic and potentially reversible regulation of gene expression, but the extent and mechanism of reversibility differ substantially among fungal lineages. In methylation-rich filamentous ascomycetes, DNA methylation is primarily associated with repeat/transposon silencing and constitutive heterochromatin organization, whereas in many other fungi, DNA methylation is reduced or absent, and analogous silencing functions are mediated largely by alternative chromatin-based systems [130,138]. In parasitic fungi, reversible methylation remodeling may facilitate stage-specific expression of effectors, stress-responsive genes, and antifungal tolerance programs. In commensal or mutualistic fungi, emerging evidence likewise suggests that methylation-based regulation can contribute to transcriptional plasticity and environmentally responsive niche adaptation, although direct mechanistic evidence remains more limited and lineage-specific [152,153]. DNA methylation dynamics may also intersect with conidiation, sporulation, and dimorphic transitions by modulating developmental gene accessibility. From an applied perspective, programmable methylation and demethylation are increasingly relevant to fungal strain engineering and the activation or repression of biosynthetic pathways without introducing permanent DNA sequence changes.

### 3.2. The Dynamics of Histone Modifications

Eukaryotic DNA is organized into chromatin via histone octamers forming nucleosomes. Histone N-terminal tails and core domains undergo diverse post-translational modifications (PTMs), including acetylation, methylation, phosphorylation, ubiquitination, SUMOylation, acylation, and ADP-ribosylation, which regulate nucleosome stability and effector protein recruitment [154,155]. Growing evidence indicates that many histone PTMs exhibit rapid turnover and spatial dynamics. Genome-wide and time-resolved studies show that acetylation and some methylation marks can be reversibly established within minutes in response to signaling and transcriptional cues [156,157,158]. Ongoing nucleosome turnover and repositioning continually reshape chromatin, generating PTM patterns that reflect a dynamic balance between enzymatic activity and chromatin structure [159]. Consequently, during development, stress, and DNA damage, histone PTM landscapes are extensively remodeled yet often partially reversible, contributing to phenotypic plasticity without permanent genetic change [160,161].

In fungi, chromatin organization and its regulation by histone PTMs are increasingly recognized as highly dynamic processes that support reversible regulatory responses and phenotypic state transitions under fluctuating internal and external conditions. Unlike sequence-level changes that often persist once established, histone marks including acetylation, methylation, phosphorylation, and ubiquitination can be reversibly deposited and removed by dedicated writer and eraser enzymes, respectively (Figure 2b) [155,162]. This epigenetic plasticity allows fungi to rapidly reprogram gene expression in response to environmental stresses such as nutrient limitation, oxidative stress, and host immune pressure, thereby facilitating phenotypic switching without alterations to the underlying DNA sequence. Importantly, in many cases, basal epigenetic states can be restored following the removal of the inducing stimuli [155,163]. Although the dynamic regulation of histone modifications has been extensively characterized in yeast and mammalian systems, related reversible chromatin-based mechanisms have been implicated in fungal morphogenesis, virulence-associated gene expression, and secondary metabolite biosynthesis in species-specific contexts [144,163].

Histone lysine acetylation is among the most dynamically regulated post-translational modifications in fungi. Histone acetyltransferases (HATs) catalyze lysine acetylation on histone tails, neutralizing positive charges, weakening DNA–histone interactions, increasing chromatin accessibility, and generally promoting transcriptional activation. Conversely, histone deacetylases (HDACs) remove acetyl groups, resulting in chromatin compaction and transcriptional repression [164,165]. This reversible acetylation–deacetylation switch enables rapid transitions between permissive and repressive chromatin states in response to environmental cues, such as carbon source shifts or antifungal exposure. In *Saccharomyces cerevisiae*, derepression of the glucose-repressed ADY2 gene upon carbon source changes requires Snf1-dependent recruitment of the HAT Gcn5 and rapid enrichment of histone H3 acetylation at its promoter, whereas under glucose-repressing conditions, the class I HDAC Rpd3 maintains a hypoacetylated, repressive chromatin state at glucose-responsive promoters [166]. Similarly, in *Candida albicans*, genetic or pharmacological inhibition of the H3K56 deacetylase Hst3 leads to accumulation of H3K56 acetylation, increased promoter occupancy at adhesion and virulence associated genes, and their elevated transcription, while normal Hst3 activity constrains their expression [167]. Consistent with this mechanism, the antifungal compound shikonin and the deacetylation inhibitor nicotinamide enhance H3K56 acetylation and sensitize *Candida. albicans* to antifungal treatment, illustrating how HDAC inhibition drives a hyperacetylated, drug-sensitive transcriptional state [168]. Nevertheless, complete characterization of stress-induced histone acetylation followed by deacetylation upon stress relief remains lacking.

Fungal chromatin is also heavily decorated by conserved histone H3 methylation marks with distinct regulatory roles. H3K4 mono-, di-, and trimethylation are enriched at transcriptionally active promoters, whereas H3K9 methylation marks constitutive heterochromatin associated with stable gene repression. In a lineage-dependent manner, H3K27 methylation mediates facultative silencing, while H3K36 methylation localizes to gene bodies and correlates with transcriptional elongation [169,170]. In filamentous fungal pathogens, methylation of these residues influences development, secondary metabolism, virulence gene expression, host-interaction-associated transcriptional programs, and environmental stress responses [163,170,171]. Their dynamic nature is maintained by histone methyltransferases and demethylases, enabling reversible chromatin states. A well-characterized example is found in *Schizosaccharomyces pombe*, where H3K9 dimethylation defines facultative heterochromatin that balances transcriptional repression with regulatory flexibility. Experimental evidence demonstrates that reversible heterochromatin formation can modulate drug resistance gene expression (Figure 2b) [8,172]. For instance, drug-induced heterochromatic islands formed through H3K9 methylation lead to the epigenetic silencing of resistance-associated genes, and both the heterochromatin state and the resulting resistant phenotype are lost upon withdrawal of the selective pressure, indicating reversibility in the studied system [8,173]. Similar mechanisms have been reported across fungal and mammalian systems, indicating that heterochromatin-mediated repression functions as a reversible epigenetic regulator of drug-response pathways [172,174]. This epigenetic plasticity enables fungal populations to tolerate transient stress without permanent genetic change, supporting a bet-hedging survival strategy.

Histone modifications do not always function in isolation but instead may operate in concert with other epigenetic regulatory systems. For example, histone H2B ubiquitination (H2Bub) has been identified in multiple fungal species, where it acts as an upstream regulatory signal for subsequent histone methylation events, including H3K4 and H3K79 methylation, thereby contributing to transcriptional regulation [175,176]. Although direct mechanistic studies in fungi remain relatively limited, the core pathways governing reversible ubiquitination and deubiquitination are evolutionarily conserved across eukaryotes [177,178]. In certain fungal species, H3K9 methylation has been shown to recruit DNA methyltransferases, thereby coupling dynamic histone modifications with more stable DNA methylation patterns [131,141]. Moreover, ATP-dependent chromatin remodelers interpret histone modification signals to reposition nucleosomes, generating feedback loops that reinforce environmentally responsive chromatin states [179,180]. Collectively, elucidating the dynamic interplay among histone modifications and other epigenetic mechanisms provides a conceptual framework for the development of reversible antifungal strategies and targeted fungal strain engineering. These reversible histone-based switches are also directly relevant to fungal ecology. In parasitic interactions, they can transiently reprogram virulence-associated loci and antifungal response pathways; in commensal or mutualistic settings, they may fine-tune nutrient acquisition, stress buffering, and partner compatibility. Histone PTM dynamics further intersect with conidiation, sporulation, and dimorphic switching by controlling transcriptional thresholds at developmental regulators. Consequently, histone writers, erasers, and readers are not only central to fungal stress biology but are also emerging as valuable targets for antifungal intervention and as tools for reversible control of industrially important traits.

### 3.3. Chromatin Remodeling

Chromatin remodeling refers to the ATP-dependent repositioning, eviction, or structural reorganization of nucleosomes, thereby modulating DNA accessibility required for transcription, DNA repair, replication, and other DNA-templated processes. In fungi, chromatin architecture is highly dynamic, and chromatin remodeling complexes actively regulate nucleosome positioning and composition in response to environmental cues, developmental signals, or cellular stress [169,181,182]. Through these coordinated activities, chromatin remodelers enable rapid and reversible alterations in gene expression programs and associated physiological states. Given the inherently dynamic nature of nucleosome displacement and repositioning, chromatin remodeling represents a major contributor to epigenetic plasticity, allowing fungi to undergo reversible phenotypic state changes without alterations to the underlying DNA sequence.

ATP-dependent chromatin remodelers have been extensively characterized in yeasts and pathogenic filamentous fungi. Members of the SWI/SNF family, including the SWI/SNF and RSC complexes, utilize the energy derived from ATP hydrolysis to slide, evict, or restructure nucleosomes, thereby modulating DNA accessibility at promoters and enhancers [183,184]. Systematic analyses in *Saccharomyces cerevisiae* and other fungal species have identified conserved subunit compositions and demonstrated that these complexes regulate transcription initiation, cell-cycle progression, and chromosomal stability [182,185,186]. In pathogenic fungi, SWI/SNF and RSC complexes have been further implicated in the regulation of hyphal differentiation, biofilm formation, stress responses, and virulence, thereby linking chromatin remodeling to environmentally responsive phenotypic transitions in species where these roles have been described [186,187]. Beyond the SWI/SNF family, additional remodeler families and histone variants also contribute to reversible chromatin-mediated regulation and phenotypic plasticity. The SWR1 complex and related remodelers catalyze the exchange of canonical histone H2A with the variant H2A.Z, generating specialized nucleosomes that prime promoters for rapid transcriptional activation or repression. Consistent with this model, focused reviews in fungal systems indicate that H2A.Z deposition and removal at stress-responsive and developmental genes fine-tune transcriptional outputs under fluctuating environmental conditions [188,189,190].

Chromatin remodeling, in concert with histone modifications and heterochromatin formation, establishes reversible chromatin states. In *Magnaporthe oryzae*, the RSC1 remodeler promotes open chromatin and restricts H3K27me3 spreading at effector and metabolic genes, thereby coordinating transcription during host infection [191]. In *Saccharomyces cerevisiae*, ATP-dependent remodelers facilitate Sir2-mediated histone deacetylation at the HML and HMR loci, generating transcriptionally silent yet reversible chromatin states [192,193]. Similarly, in *Schizosaccharomyces pombe*, the Clr4-dependent H3K9 methylation pathway works with chromatin remodelers like Swi6-associated complexes to establish heterochromatin domains that are stably inherited yet reprogrammable under specific conditions [194]. Thus, chromatin remodelers and histone-modifying enzymes generate dynamic chromatin landscapes that enable reversible gene expression regulation without altering the underlying DNA sequence.

In summary, chromatin remodeling functions as a central regulatory axis through which fungi dynamically reshape nucleosome architecture to interpret environmental signals and modulate gene expression without altering genomic sequence. By integrating ATP-dependent remodelers, histone-modifying enzymes, and heterochromatin pathways, fungal cells generate flexible epigenetic landscapes that enable rapid and reversible transitions between physiological states. This coordinated remodeling capacity is integral not only to stress responses, morphogenesis, and secondary metabolism, but also to conidiation, sporulation, and dimorphic transitions that allow fungi to cope with adverse conditions. Accordingly, chromatin remodelers are increasingly viewed as important mediators of fungal phenotypic flexibility and as tractable tools for reversible gene regulation in biotechnology.

### 3.4. Noncoding RNAs

Unlike protein-coding RNAs, noncoding RNAs (ncRNAs) do not encode proteins but are characterized by rapid synthesis and turnover, enabling dynamic and transient regulation of gene expression and chromatin states. Accordingly, ncRNAs are increasingly recognized as key mediators of phenotypic plasticity in fungi, contributing to diverse biological processes, including development, metabolism, stress responses, pathogenicity, and antifungal drug responses [195,196]. Although early genomic studies primarily focused on protein-coding genes, transcriptome-wide approaches, such as RNA sequencing (RNA-seq) and tiling array analyses, have revealed the widespread presence of noncoding transcripts, even in microorganisms previously assumed to possess highly compact genomes [197].

Fungal ncRNAs constitute a versatile regulatory toolkit that operates at multiple levels—including RNA stability, transcription, and chromatin state—to enable dynamic gene expression control. A large fraction of fungal long non-coding RNAs (lncRNAs) arises from antisense or intergenic transcription. In model yeasts such as *Saccharomyces cerevisiae* and *Schizosaccharomyces pombe*, tiling array and RNA-seq studies have revealed pervasive antisense transcription, with noncoding RNAs frequently overlapping or undergoing antisense transcription to annotated genes, suggesting regulatory mechanisms involving transcriptional interference or chromatin-associated factors [197,198]. Supporting this view, a comparative analysis of five major *Candida* pathogens identified hundreds of lncRNAs, many co-expressed with protein-coding genes, while some show infection-stage-specific expression, implying roles in stress responses or host-associated regulation [199]. Fungal lncRNAs are increasingly linked to epigenetic regulation, including recruitment of chromatin-modifying or -remodeling complexes, modulation of nucleosome positioning, and alteration of histone modification patterns, thereby affecting transcriptional accessibility [195,196]. In fungi retaining RNA interference (RNAi) machinery, small RNAs derived from double-stranded RNAs or structured ncRNAs mediate post-transcriptional gene silencing, enabling rapid responses to environmental stress or antifungal drugs [196,200]. Additionally, in some plant pathogenic fungi such as powdery mildew species, antisense transposable element-derived lncRNAs dynamically regulate transposons and adjacent genes during infection, contributing to transcriptional plasticity and host adaptation [201,202].

NcRNAs are therefore best considered a dynamic epigenetic regulatory layer whose functions are highly species- and context-dependent. In pathogenic fungi, ncRNA-mediated regulation has been implicated in antifungal drug responses, morphogenetic switching, stress adaptation, and context-dependent virulence. In broader ecological settings, emerging evidence further suggests that fungal ncRNAs can contribute to metabolic plasticity and environmentally responsive transcriptional tuning under changing abiotic conditions [196,202]. Their rapid inducibility and reversibility also make them relevant to developmental transitions, including sporulation and dimorphism, where transient reprogramming of chromatin and transcription is essential. These same properties make ncRNA-based systems attractive for biotechnology, because they offer opportunities to modulate fungal phenotypes reversibly without permanently rewriting the genome.

### 3.5. RNAi

RNA interference (RNAi) is a conserved regulatory mechanism that employs small non-coding RNAs to mediate gene silencing at transcriptional and post-transcriptional levels (Figure 2c). In fungi, RNAi supports reversible regulatory responses to environmental stress, development, and genome defense, but its retention and mechanistic deployment differ substantially among taxa [203,204,205]. Canonical RNAi-linked heterochromatin assembly has been most clearly resolved in the ascomycete *Schizosaccharomyces pombe*, whereas filamentous ascomycetes such as *Neurospora crassa* have revealed an expanded diversity of RNAi-related pathways [205,206,207]. By contrast, some fungal lineages have partially or completely lost RNAi, illustrating that reversible RNA-based regulation is phylogenetically uneven rather than universal

Fungal RNAi is mediated by a conserved core machinery consisting of Dicer, Argonaute, and RNA-dependent RNA polymerase (RdRP). Double-stranded RNA derived from transposable elements, repetitive regions, aberrant transcripts, or exogenous sources is processed by Dicer into small RNAs, which are incorporated into Argonaute-containing silencing complexes to direct sequence-specific repression at transcriptional or post-transcriptional levels. RdRPs further amplify RNAi by generating secondary dsRNA [203,204,205]. In *Neurospora crassa*, multiple specialized RNAi-related pathways have been described, including quelling [208], meiotic silencing by unpaired DNA (MSUD) [209], and DNA-damage-induced qiRNAs [210]. Quelling represents a post-transcriptional gene-silencing mechanism triggered by repetitive transgenes and has been foundational both for understanding fungal RNA biology and for developing reverse-genetic approaches in fungi [210]. In *Schizosaccharomyces pombe*, by contrast, RNAi operates prominently through the RITS complex, which couples small RNAs to H3K9 methylation and heterochromatin formation at centromeric and other repetitive regions [206]. Together, these examples show that fungal RNAi is not a single pathway but a family of context-dependent mechanisms linking RNA surveillance, chromatin regulation, and phenotypic plasticity. A well-characterized example of reversible RNA-based phenotypic regulation is provided by epimutation in *Mucor circinelloides* [7,211,212]. Exposure to FK506 selects drug-resistant epimutants in which fkbA-specific small RNAs silence the FKBP12-encoding gene without underlying DNA mutation. Removal of drug pressure reduces small RNA production, restores fkbA expression, and returns cells to a drug-sensitive state, demonstrating a reversible RNAi-mediated mechanism of antifungal drug-response plasticity. Similar RNAi-dependent epimutations can also confer resistance to rapamycin and 5-fluoroorotic acid, and many of these states revert in drug-free environments, consistent with an RNAi-based bet-hedging strategy. In host environments, the organ-specific stability and reversion of such epimutations further suggest that local conditions can modulate RNAi activity and thereby tune fungal phenotypes during infection.

Beyond drug-response plasticity, RNAi pathways also contribute to fungal ecology and development. In parasitic interactions, RNAi can regulate transposons, effectors, and stress-response genes, thereby supporting host adaptation and immune evasion. In broader ecological settings, RNA-based silencing is likely to contribute to metabolic flexibility and environmentally responsive gene regulation, although direct mechanistic evidence remains uneven across fungal lifestyles [203,204,205]. RNAi is also relevant to conidiation, sporulation, and dimorphic transitions, because many of these developmental programs require rapid but reversible adjustment of transcriptional networks and chromatin states [200,203]. The classical *Neurospora* pathways and the *Schizosaccharomyces pombe* RITS system illustrate how RNAi can act either through transcript degradation or through chromatin-based silencing to coordinate such responses [206,208,209,210].

The conceptual and practical significance of fungal RNAi extends well beyond basic biology. From a clinical perspective, RNAi-dependent epimutations imply that some forms of antifungal resistance may be transient and therefore susceptible to therapeutic strategies targeting RNAi or chromatin-based regulation. From an experimental perspective, quelling and related pathways have already been exploited for gene-function analysis and reversible gene knockdown. In biotechnology and agriculture, engineered RNAi systems and exogenous double-stranded RNA have been used to modulate virulence, toxin production, and developmental traits without permanently modifying the genome. RNAi therefore represents one of the clearest examples of how fungi use reversible epigenetic mechanisms to balance genome stability with rapid environmental responsiveness [203,204,211,213].

### 3.6. Integrative Perspective

Taken together, reversible epigenetic changes in fungi emerge from the combined action of DNA methylation, histone modification, chromatin remodeling, ncRNAs, and RNAi-related pathways such as quelling and RITS (Table 1). However, the degree and timescale of reversibility differ substantially among mechanisms, species, and environmental contexts. Their distribution is uneven across fungal lineages, with several canonical mechanisms best characterized in ascomycetes, although functionally analogous principles may operate more broadly. Across parasitic, commensal, and mutualistic interactions, these systems allow fungi to modulate host interaction, metabolism, stress tolerance, and developmental timing without requiring immediate permanent genetic change. Their repeated involvement in sporulation, conidiation, and dimorphic transitions underscores that reversibility is tightly integrated with fungal survival strategies under fluctuating environments. These same properties make epigenetic mechanisms especially attractive for antifungal intervention and for non-permanent engineering of industrial or agricultural fungal traits.

**Table 1:** Reversible genetic and epigenetic mechanisms contributing to phenotypic variability in fungi.

Mechanism	Representative Species	Major Fungal Lineage(s)	Principal Mode	Biological Relevance/Application
Mini-/supernumerary chromosomes	*Fusarium oxysporum*, *Candida albicans*	Ascomycetes	Gain/loss of dispensable chromosomes; gene dosage change	Host specificity, virulence, antifungal tolerance, chromosome engineering
Transposable elements	*Magnaporthe oryzae*, *Zymoseptoria tritici*, multiple fungi	Both	Stress-responsive mobilization/silencing	Effector diversification, genome plasticity, strain improvement
Mating-type switching	*Saccharomyces cerevisiae*, *Schizosaccharomyces pombe*	Ascomycetes	Programmed locus replacement/imprinting- dependent switching	Reproductive assurance, sporulation under nutrient stress
Tandem repeats/MTD	Model yeasts and filamentous fungi	Predominantly ascomycetes	Repeat expansion/contraction; reversible gene dosage modulation	Drug tolerance, stress adaptation, metabolic tuning
RIP	\	Ascomycetes	Premeiotic C:G-to-T:A transition mutation of duplicated DNA	Genome defense, TE control, genome stabilization
MIP	*Ascobolus immersus*	Ascomycetes	Premeiotic methylation of duplicated DNA	Repeat silencing, genome defense, epigenetic regulation
Heterokaryosis	Filamentous fungi, including plant pathogens and symbionts	Both	Coexistence of genetically distinct nuclei in one cytoplasm	Rapid phenotypic buffering, host adaptation, parasexuality
Prion inheritance	*Saccharomyces cerevisiae*	Ascomycetes	Protein-based heritable conformational switching	Stress adaptation, translational rewiring, phenotypic diversification
RNA mutagenesis/RNA editing-like transcript diversification	Multiple fungi	Both	Transient transcript-level variation without stable DNA change	Rapid environmental response, host interaction, regulatory plasticity
DNA methylation/demethylation	*Neurospora crassa*, *Magnaporthe oryzae*, *Cryptococcus neoformans*	Both, lineage- dependent-	Dynamic DNA methylation remodeling	TE repression, developmental control, pathogenicity
Histone modifications	*Saccharomyces cerevisiae*, *Candida albicans*, *Schizosaccharomyces pombe*	Both	Reversible chromatin-state transitions	Virulence regulation, antifungal response, morphogenesis
Chromatin remodeling/H2A.Z exchange	*Saccharomyces cerevisiae*, *Magnaporthe oryzae*	Both	ATP-dependent nucleosome repositioning/variant exchange	Effector regulation, stress responses, developmental transitions
ncRNAs/RNAi/quelling/RITS	*Neurospora crassa*, *Mucor circinelloides*, *Schizosaccharomyces pombe*	Both, pathway-specific	Post-transcriptional and transcriptional silencing	Drug epimutation, heterochromatin formation, biotechnology

## 4. Conclusions

Fungi exemplify how phenotypic plasticity and regulatory flexibility can make important contributions to survival and persistence under fluctuating environmental conditions. Rather than relying solely on rare, irreversible genetic change, fungi deploy a layered set of reversible genetic and epigenetic mechanisms that allow rapid adjustment to fluctuating environments while preserving core genome integrity. Processes such as transposable element regulation, tandem repeat variation, mating-type switching, and the dynamic gain or loss of accessory chromosomes provide flexible control over genome architecture and gene dosage, enabling reversible phenotypic variation and state transitions without long-term commitment. Superimposed on this genetic flexibility, epigenetic systems—including histone modifications, DNA methylation, chromatin remodeling, noncoding RNAs, and RNA interference—translate environmental signals into transient yet heritable transcriptional states. These mechanisms allow fungi to rapidly tune programs governing stress tolerance, metabolism, development, and pathogenicity on timescales that may be incompatible with classical mutation-driven evolution. Importantly, a substantial subset of these responses is reversible or decays after the removal of selective pressure, although the extent of reversibility varies across mechanisms, species, and environmental contexts, thereby potentially limiting some fitness trade-offs. Together, these observations support a unified view in which fungi’s success in survival across diverse and often extreme environments is supported in part by selection for reversible regulatory and genetic plasticity. From this perspective, traits such as drug resistance or virulence may be best understood not as fixed endpoints but as context-dependent outcomes emerging from heterogeneous and dynamically regulated states. Looking forward, integrating long-read genomics, single-cell epigenomics, and live-cell imaging will be essential to resolve how these reversible systems are coordinated, with important implications for antifungal strategies, biotechnology, and agricultural management.

## Data Availability

No new data were created or analyzed in this study. Data sharing is not applicable to this article.
